# Feed supplementation with biochar may reduce poultry pathogens, including *Campylobacter hepaticus*, the causative agent of Spotty Liver Disease

**DOI:** 10.1371/journal.pone.0214471

**Published:** 2019-04-03

**Authors:** Nicky-Lee Willson, Thi T. H. Van, Surya P. Bhattarai, Jodi M. Courtice, Joshua R. McIntyre, Tanka P. Prasai, Robert J. Moore, Kerry Walsh, Dragana Stanley

**Affiliations:** 1 Central Queensland University, Institute for Future Farming Systems, Rockhampton, Queensland, Australia; 2 The University of Adelaide, School of Animal and Veterinary Sciences, Roseworthy, South Australia, Australia; 3 RMIT University, School of Science, Bundoora, Victoria, Australia; 4 University of Southern Queensland, Toowoomba, Queensland, Australia; 5 Department of Livestock Services, Ministry of Agriculture, Land Management and Cooperatives, Government of Nepal, Hariharbhawan, Lalitpur, Nepal; University of Connecticut, UNITED STATES

## Abstract

Increased global regulation and restrictions on the non-therapeutic use of antibiotics in the poultry industry means that there is a need to identify alternatives that prevent infection while still conveying the growth and performance benefits afforded by their use. Biochars are produced by the incomplete pyrolysis of organic materials, with reports of use as a feed supplement and activity against pathogenic bacteria. In the current study the dose-dependent effects of biochar dietary inclusion in layer diets at 1%, 2% and 4% w/w were investigated to determine a) the efficacy of biochar as an anti-pathogenic additive on the intestinal microbiota and b) the optimal inclusion level. Biochar inclusion for anti-pathogenic effects was found to be most beneficial at 2% w/w. Poultry pathogens such as *Gallibacterium anatis* and campylobacters, including *Campylobacter hepaticus*, were found to be significantly lower in biochar fed birds. A shift in microbiota was also associated with the incorporation of 2% w/w biochar in the feed in two large scale trials on two commercial layer farms. Biochar inclusion for anti-pathogenic effects was found to be most beneficial at 2% w/w. Differential effects of the timing of biochar administration (supplementation beginning at hatch or at point of lay) were also evident, with greater impact on community microbial structure at 48 weeks of age when birds were fed from hatch rather than supplemented at point of lay.

## Introduction

Supplementation of antibiotics in animal feed has been widespread in the global poultry industry for over 60 years, reducing the load of pathogenic bacteria and improving growth and feed conversion efficiency [1]. Antibiotic use in livestock production is projected to increase by 67% between 2010–2030 if no action is taken in currently unregulated developing countries [2]. Concern over the emergence of antibiotic resistant pathogens has led to the banning of non-therapeutic antibiotic use in livestock production in Europe in 2006 [3, 4]. Further tightening of regulations governing antibiotic use is likely in other jurisdictions worldwide, with the Food and Agriculture Organization of the United Nations (FAO), the World Health Organization (WHO) and the World Organization for Animal Health collaborating to address antimicrobial resistance [5, 6]. Thus, the poultry industry needs to identify alternatives that reduce pathogen loads while still conveying the growth and performance benefits associated with the in-feed use of antibiotics. Alternatives are also needed for organic producers and the layer industry, which cannot routinely use in-feed antibiotics due to residue carry over to the egg.

Biochar is produced by the incomplete pyrolysis (heating to ~550°C under oxygen limited conditions) of organic materials such as wood, straw, manure, crop residues and leaves [7]. Depending on feed material and pyrolysis condition, biochar contains (on a w/dw basis) 40–80% carbon, 0.1–0.8% nitrogen, 1–2% potassium, 5–6% calcium and can have an ion exchange capacity between 25 and 150 cmol^+^/kg [7]. There are many potential uses of biochars, e.g. as a mineral fertiliser or as a soil ameliorant for improving soil water holding capacity and/or ion exchange capacity, as a recalcitrant carbon store, and as an adsorbent of organic toxins and other compounds [7, 8]. Biochars have been applied to poultry litter management, functioning to reduce free moisture content and ammonia production [9]. Biochar differs from activated carbon/charcoal, which is completely oxidised carbon ‘activated’ at high temperatures (> 700°C) using steam or chemicals. The activation process increases effective surface area by removal of residues. This process results in a material with no ionic charge but high adsorptive properties [7]. The distinction between charcoal and biochar can be blurred in the literature, with the terms often used in the context of intended use; i.e. if oxidised organic material is burnt as fuel it is referred to as charcoal, or, if used for carbon sequestration or as a soil ameliorant it is termed biochar [7, 10, 11]. More rigour in the use of these terms is needed, reserving the use of the term charcoal to completely oxidised carbon, and biochar to incomplete oxidation. The often unspecified manufacturing conditions makes literary comparisons difficult, however the use of several combustion products as in-feed additives in poultry (in the context defined for biochar) have been reported.

Poultry litter ash (PLA; the mineral ash remaining after complete combustion of litter) has been recommended as a cost-effective alternative to di-calcium phosphate [12]. Broilers fed activated coconut shell charcoal (unspecified pyrolysis conditions) had significant improvements in feed conversion ratio (FCR) during the finisher stage, particularly at an inclusion dose of 0.5% w/w, but effects were reversed with inclusion of 2% w/w or higher levels [13]. Supplementation of broiler feed with either charcoal (unspecified pyrolysis conditions) from maize cob or *Canarium schweinfurthii* Engl. fruit seeds also revealed a dose dependent response, with dietary inclusion at 0.2, 0.4 and 0.6% w/w resulting in increased final bodyweights compared to control birds, and decreased bodyweights at 0.8 and 1% inclusion [14]. Layer diet supplementation with wood oak charcoal (unspecified pyrolysis conditions) amended to 0, 1, 2 or 4% w/w inclusion was shown to increase shell thickness and decrease the incidence of egg shell cracking, but also slightly reduced feed intake, FCR and egg production in layers [15]. The direct functional mechanism of supplementation driving improvements is unclear and likely multi-factorial, however it is thought to aid in digestion and improve feed efficiency as well as bind toxins such as mycotoxins [9].

With recent advances in development of species-specific genetic markers in combination with sequencing technology advancements, we have witnessed a rise of the “golden age of microbial ecology” [16]. Significant advancements have been made in the area of poultry intestinal microbial communities and the major role they play in poultry performance, immunity, response to pathogens and intestinal homeostasis (reviewed in Oakley et al. [16] and Stanley et al. [17]). It is now well established that pathogen control starts from the hatch day with control of the initial bird colonisation and promoting the establishment of healthy intestinal microbiota with high beneficial to pathogenic bacteria ratios.

While there are limited studies on the effects of biochar supplementation in poultry on performance traits, less is known about the effects of biochar on the intestinal microbiota. Prasai et al. [18] supplemented layer pullet diets with 4% w/w biochar (produced from woody green waste by pyrolysis at 550°C) for 23 weeks and found that inclusion did not influence overall richness, diversity or community structure of the microbiota. However, a reduction in the abundance of the phylum Proteobacteria was noted with two major classes altered. Of these, an operational taxonomic unit (OTU) with 100% sequence identity across the amplified region to *Campylobacter jejuni* subsp. *jejuni* NCTC 11168 = ATCC 700819 strain, was significantly reduced from 1.3% relative abundance in control birds to 0.02% in those supplemented with biochar. *Helicobacter* were also reduced, although not significantly. Watarai and Tana [19] reported that the addition of Nekka-Rich (a product combining activated charcoal and wood vinegar liquid, both from the bark of an evergreen oak) to the diets of White Leghorns effectively inhibited the colonisation of *Salmonella enterica* serovar Enteritidis in a challenge model. The authors also demonstrated enhanced growth of *Enterococcus faecium* and *Bifidobacterium thermophilum*, both beneficial to the host. In summary, previous studies have indicated that biochars could help to control pathogen loads in poultry without significantly altering the gut microbiota [20].

In the current study, we investigated the dose-dependent effects of biochar dietary inclusion in layer diets to determine optimal inclusion level in the context of the efficacy of biochar as an anti-pathogenic additive on the intestinal microbiota of laying hens. We also report on the microbiota results from two large scale feeding trials incorporating 2% w/w biochar vs control diets on two commercial layer farms.

## Materials and methods

### Trial 1—Dose-dependent study (0, 1, 2 and 4% biochar inclusion)

All procedures involving the use of animals were approved and monitored by the Animal Ethics Committee of Central Queensland University (Approval #A 12/06-283). This initial study was performed at Central Queensland University animal house. Birds were sampled alive via cloacal swab.

Eighty Bond Brown layer pullets (17 weeks old; Bond Enterprises P/L, Grantham, QLD, Australia) were housed in a commercial layer caging system, as previously described by Prasai et al. [21] at the Central Queensland University, Rockhampton, QLD, Australia. Birds were randomly assigned to cages in a randomised block layout (4 treatments; 5 birds per cage/treatment, 4 replicates; *n* = 80). After placement, birds were housed for one week to adapt to the new environment. This age point was selected as it is a common age for commercial farms to acquire new laying pullets. Dietary treatments began thereafter and continued for a period of 23 weeks. Dietary treatments included a control diet, and three diets with biochar supplementation at 1, 2 and 4% w/w. The control diet was a standard commercial layer crumble ration (Blue Ribbon Stock Feeds, Rockhampton, QLD) comprised of: 90.4% w/w dry matter, 11.75 MJ/kg metabolisable energy, 17.9% w/w crude protein, and calcium 4.2% w/w (dry weight basis), and the composition of the biochar amended treatments was adjusted to maintain these attributes [21]. The biochar was sourced from Pacific Pyrolysis Pty Ltd (Sydney, NSW), made from woody green waste by pyrolysis at 550°C. The biochar contained 76.1% carbon, 3.16% hydrogen, 0.29% nitrogen and 0.03% sulfur (w/dw basis) with an effective cation exchange capacity of 29.7 cmol^+^/kg. At 23 weeks post dietary commencement, cloacal samples were collected from 80 birds, 20 per treatment, using sterile swabs and stored at– 80°C for analysis. DNA extraction from swabs, amplification and quality sequencing was successful on 51 birds and therefore microbiota analysis was performed on 10 samples from control, 12 from 1% biochar, 16 from 2% and 13 from 4% biochar fed birds. Performance parameters have been previously reported by Prasai et al. [22] and are not re-presented within the current manuscript. In brief, there was no significant effect on egg yield per bird, but egg mass was increased for the 2% w/w biochar treatment, and FCR was improved in all biochar supplementation groups compared to control birds.

### Trial 2 –Farm 1

All procedures involving the use of animals were approved and monitored by the Animal Ethics Committee of Central Queensland University (Approval #A 14/10-319). This study was performed at a commercial farm with their permission. Observational data and bird sampling via cloacal swab were collected by the farm employed poultry veterinarian without harming the birds.

A 20,000-bird capacity slat floor layer shed was divided into two sections (A and B) on a commercial layer farm near Brisbane, Queensland, Australia (termed Farm 1). Lohman-Brown day-old chicks were sourced from Specialised Breeders Australia P/L (Bendigo, Victoria) and were reared in a separate Farm location to 15 weeks of age (following commercial farm practice), then transferred on site to a laying shed and separated into section A (9,287 birds) and section B (9,344 birds). Layers had *ad libitum* access to feed and water. Dietary treatments included a control diet and a biochar supplemented diet at 2% w/w. The control diet comprised 94.6% dry matter, ~17.2% crude protein, 4% calcium (w/dw basis) and 11.7 MJ/kg metabolizable energy. The composition of the biochar amended diet was adjusted to maintain these attributes. Section A was fed with the control diet, while section B was fed the biochar amended diet for a period of 19 weeks after the birds first arrived on farm. The biochar was produced by Mara Seeds Pty Ltd (Mallanganee, NSW) from eucalyptus hardwood by pyrolysis at ~550°C. The biochar contained 66.2% carbon, 0.68% nitrogen (w/dw basis) and had an effective cation exchange property of 29.73 cmol^+^/kg. Cloacal swabs were collected from birds at arrival to the farm (age 15-week pullets) and at 19 weeks post arrival for biochar fed and control fed birds. DNA extraction from swabs, amplification and quality sequencing was successful on 66 birds and microbiota analysis was performed on these samples, i.e. samples collected from birds at arrival (*n* = 19) and after 19 weeks on biochar amended (*n* = 25) and control (*n* = 22) feed. Performance data are not available from the commercial producer of this flock.

### Trial 3 –Farm 2

All procedures involving the use of animals were approved and monitored by the Animal Ethics Committee of Central Queensland University (Approval #A 14/10-319). This study was performed at a commercial farm with their permission. Observational data and bird sampling via cloacal swab were collected by the farm manager without harming the birds.

Two sheds were utilised on an organic free range farm near Gympie, QLD, Australia (Farm 2). Hy-Line Brown layer day-old chicks (Specialised Breeders Australia P/L, Bendigo, Victoria) were separated into two flocks on receival and placed onto two dietary treatments. After rearing to 18 weeks of age (following the commercial farm practice), each flock was transferred into two separate layer sheds. One shed was fed using a 2% w/w biochar supplemented feed (*n* = 2,010) and a second shed fed with a control diet (*n* = 10,060), with stocking densities of 14.8 and 14.9 birds/m^2^ respectively. The control diets were comprised of (on a w/dw basis): Starter crumble: 89.3% dry matter, 12.2 MJ/kg metabolisable energy, 19.2% crude protein and calcium 1.3%; Pullet grower 89.1% dry matter, 12.3 MJ/kg metabolisable energy, 17.5% crude protein and calcium 1.3%; Layer 90.1% dry matter, 11.4 MJ/kg metabolisable energy, 17.2% crude protein and 4.4% calcium. The composition of the biochar 2% w/w diets were amended to maintain these attributes. The biochar was produced by Mara Seeds Pty Ltd (Mallanganee, NSW) from eucalyptus hardwood by pyrolysis at ~550°C, as used in Trial 2.

Cloacal swabs were collected from biochar fed birds at 48 weeks of age. DNA extraction from swabs, amplification and quality sequencing was successful on 29 birds and microbiota analysis was performed on biochar (*n* = 16) and control (*n* = 13) fed birds. Performance data for these birds has previously been published by Prasai et al. [22] and subsequently not re-presented within the current manuscript. Birds receiving the 2% w/w biochar supplementation had increased egg production and resulted in no differences in egg weight. It is acknowledged that this performance data, although based on a very high number of birds, lacked shed replication.

### DNA extraction, amplification and sequencing

Sample processing was the same for all samples collected across all trials using cloacal swabs. This sampling method has previously been compared to cecal samples in poultry and found to be qualitatively similar [23]. This method allows for detection of some shifts and responses of cecal microbiota without having to sacrifice animals-which was not possible on the commercial farms. Total DNA was extracted from swabs using a Bioline ISOLATE faecal DNA kit (#BIO-52038) and run to the manufacturer’s specifications. The V3-V4 region of 16S sRNA was amplified with forward primer 5’ ACTCCTACGGGAGGCAGCAG 3’and reverse primer 5’ GGACTACHVGGGTWTCTAAT 3’ using Q5 high fidelity polymerase (New England Biolabs). Sequencing was performed on an Illumina MiSeq system (2 x 300 bp) by the method of Fadrosh et al. [24]. Sequence data was analysed using QIIME version 1.9.1 [25] using default parameters and Phred quality threshold of > 20. Uclust algorithm [26] was used to pick OTUs at 97% sequence identity. Chimeric sequences were inspected using Pintail [27]. Blast was used to assign taxonomy against the GreenGenes database [28] and QIIME version 1.9.1 defaults. Additional assignment of taxonomy was performed using a command line version of blastn [29] against the NCBI 16S Microbial database. Further visualisation and analysis of the sequence data was performed using Calypso v8.20 [30] using TSS normalisation and SquareRoot transformation.

### Statistical analyses

Three separate animal trials were analysed investigating the effects of in-feed biochar supplementation on the intestinal microbiota of poultry. Following quality control and analysis as described above, all microbiota data were normalised using TSS normalisation and SquareRoot transformation in Calypso v8.20 [30]. Trial 1 (Dose-dependent study) was a 1 x 2 (diet and pen) randomised block design and analysed by pairwise t-tests in Calypso v8.20 and a one-way ANOVA in SPSS (IBM SPSS). Pearson’s Correlations were used to determine correlations between microbiota abundance and biochar at four inclusion levels. Trial 2-Farm 1 and Trial 3-Farm 2 were both 1x1 trial designs without shed replication and analysed by t-test in Calypso v8.20. PCR results for the presence or absence of *Campylobacter hepaticus* in remaining DNA samples were compared using a Chi Square test in SPSS (IBM SPSS).

### Nucleotide sequence accession numbers

The sequence data and sample metadata have been submitted to MG-RAST database under accession number mgl716494.

### PCR for Spotty Liver pathogen carriage

The presence of the Spotty Liver Disease causative pathogen, *C*. *hepaticus*, was investigated using the PCR method as previously described by Van et al. [31], in which species-specific primers for *C*. *hepaticus* were designed and validated both in vitro and in silico. PCR was conducted on the remaining DNA extracted for the microbiota analysis of control (*n* = 16) and biochar (*n* = 13) fed birds (after 19 weeks of biochar amended feed treatment) from Farm 1, and control (*n* = 13) and biochar (*n* = 16) fed birds (after 48 weeks of biochar amended feed treatment) from Farm 2.

## Results

### Trial 1 –Dose-dependent study

**Fig 1:**
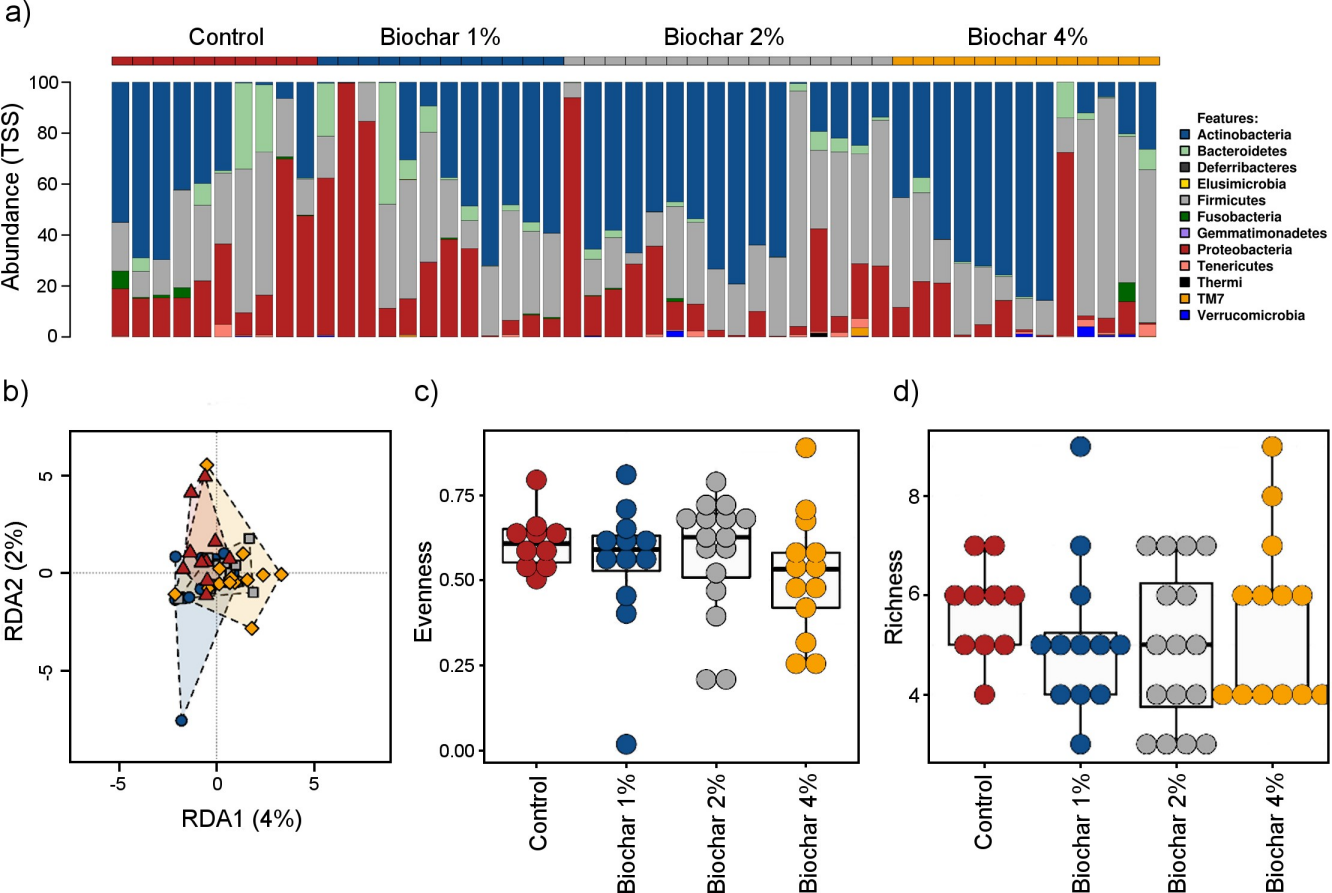
Microbial diversity in cloacal swabs of layer chickens fed for 23 weeks on control and 1%, 2% or 4% w/w biochar amended diets (Trial 1). a) Relative phylum abundance; b) Phylum level redundancy analysis (RDA) plot shows no differentiation (*P* = 0.355) between dietary treatments; c) Evenness (phylum) and d) Richness (phylum) indices were not significantly different between any dietary treatment.

The combined representative phyla (% abundance) of the microbiota in cloacal swabs of laying hens following 23 weeks of dietary treatment were Actinobacteria (39.6%), Firmicutes (31.7%), Proteobacteria (22.5%) and Bacteroidetes (4.7%). Phyla accounting for less than 1% included Verrucomicrobia, Tenericutes, Fusobacteria, and TM7 while Thermi, Elusimicrobia, Deferribacteres and Gemmatimonadetes represented less than 0.5% (Fig 1A). Representative phyla were not significantly different between the four dietary treatment groups nor did the inclusion of biochar at either 1, 2 or 4% w/w result in differences in microbiota community structure at phylum (*P* = 0.355), genus (*P* = 0.303) or species (*P* = 0.420) level as measured by Bray-Curtis similarity and permutational multivariate analysis of variance using Adonis. Lack of community differentiation at phylum level was evident in the redundancy analysis plot (Fig 1B). Diversity indices were not significant between dietary groups at either phylum (Shannon Index, *P* = 0.323; Evenness, Fig 1C, *P* = 0.569; Richness, Fig 1D, *P* = 0.571; and Chao1, *P* = 0.380) or genus level (Shannon Index, *P* = 0.529; Evenness, *P* = 0.454; Richness, *P* = 0.938, and Chao1, *P* = 0.542).

**Fig 2:**
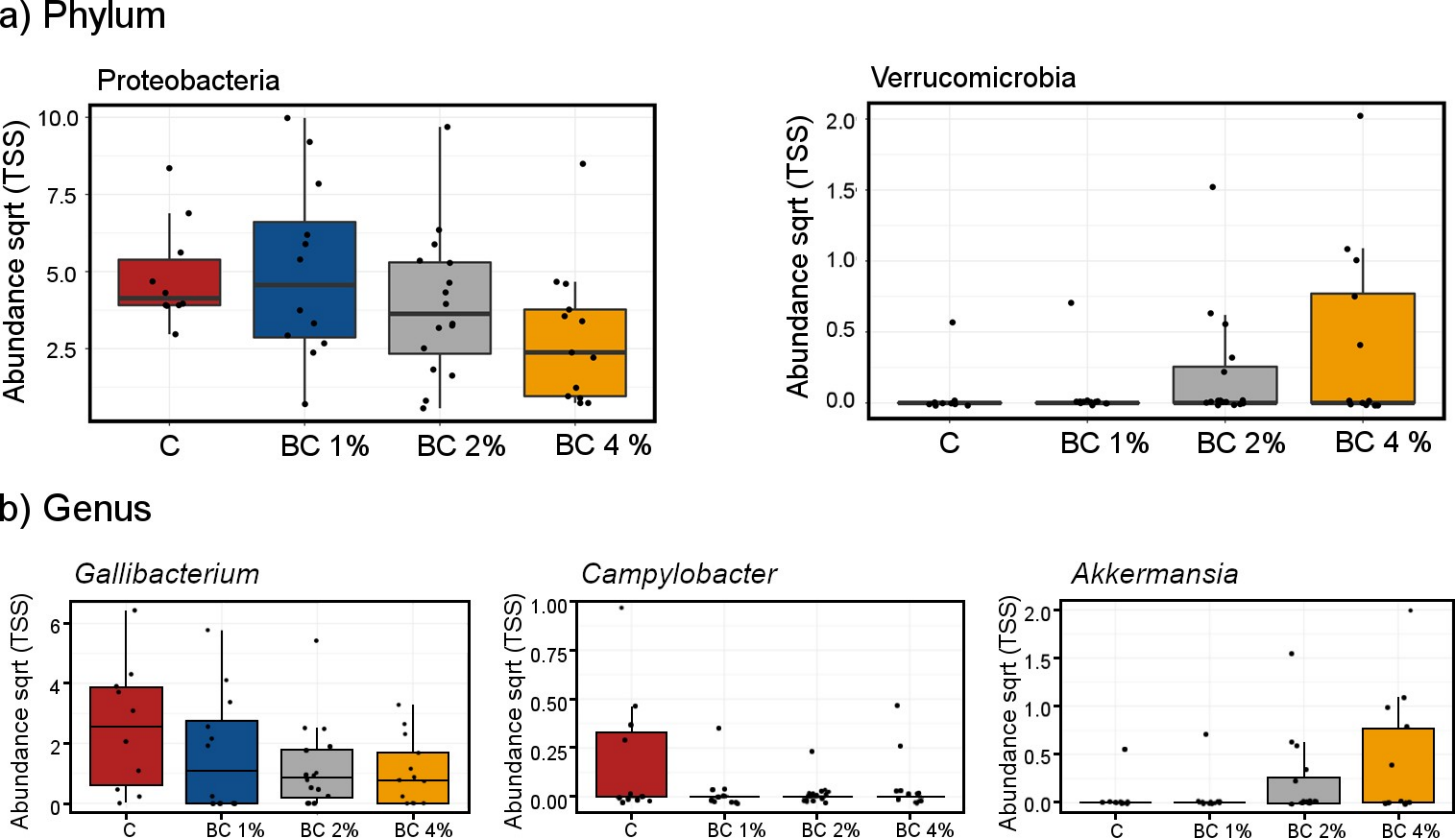
Microbial diversity in cloacal swabs of layer chickens fed for 23 weeks on control (C) and 1%, 2% or 4% w/w biochar (BC) amended diets (Trial 1). a) Phylum abundance of Proteobacteria and Verrucomucrobia and b) Genus abundance of known pathogenic *Gallibacteruim*, *Campylobacter* and the beneficial *Akkermansia*.

Pearson’s correlation based analyses of linear relationships were performed to consider the effect of increasing biochar dose on bacterial abundance. At phylum level, Proteobacteria (R = -0.33, *P* = 0.018) were negatively correlated while Verrucomicrobia (R = 0.32, *P* = 0.022) were positively correlated with increasing biochar inclusion (Fig 2A). A total of 11 OTUs were correlated with biochar dose rate. Significant negative correlations with species belonging to the phylum Proteobacteria included two from genus *Gallibacterium* (Fig 2B), *G*. *genomosp 1* (R = -0.38, *P* = 0.006) and *G*. *anatis* (R = -0.28, *P* = 0.047). Positive correlations with biochar inclusion were noted with unclassified *Paracoccus* (R = 0.32, *P* = 0.021) and unclassified *Paucibacter* (R = 0.30, *P* = 0.035). One-way ANOVA analysis of Proteobacteria demonstrated significant reductions in *Campylobacter* (*P* = 0.032; Fig 2B) at 1% and 2% w/w inclusion relative to control, however the abundance was increased slightly in the 4% w/w biochar treatment. This trend was seen for other bacterial species suggestive that lower doses were more effective in pathogen removal than the 4% w/w treatment. Two taxa belonging to the phylum Actinobacteria were significantly correlated with dose rate; unclassified *Bifidobacteriaceae* (R = 0.37, *P* = 0.007) and unclassified *Streptomyces* (R = -0.33, *P* = 0.017). An OTU, most highly similar to *Lactobacillus aviaries* across the amplified region (R = 0.33, *P* = 0.017) was positively correlated with increasing biochar dose, as was an OTU assigned to *Akkermansia muciniphila* (R = 0.32, *P* = 0.022) a known beneficial species. Fig 2B demonstrates the effects of biochar dose at the genus level for pathogenic *Gallibacterium*, and *Campylobacter*, and beneficial *Akkermansia*.

### Trial 2—Farm 1

**Fig 3:**
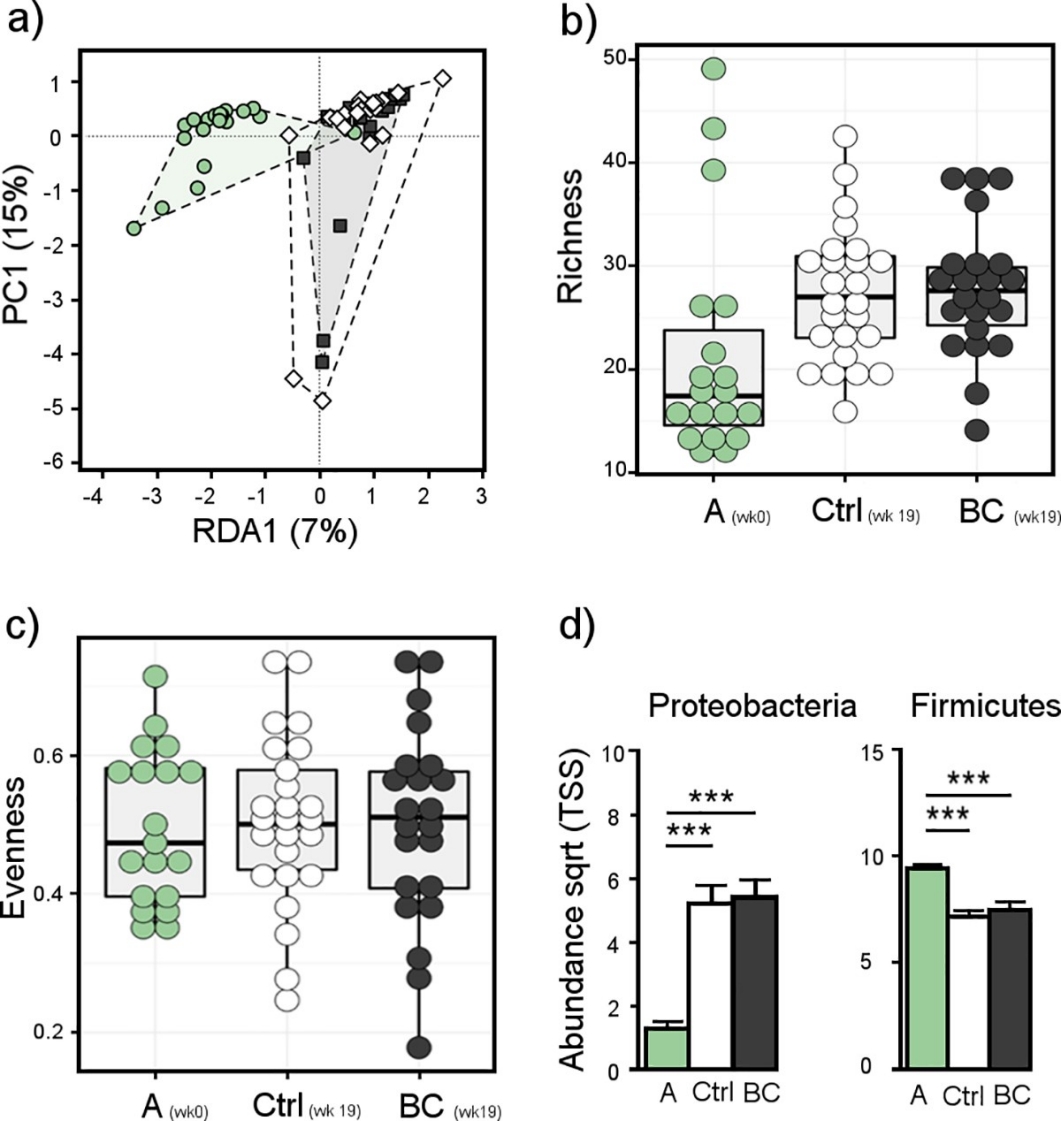
Microbial diversity in cloacal swabs of layer chickens fed on control and 2% w/w biochar amended diets for a period of 19 weeks (Trial 2) at arrival to farm (A wk 0), and 19 weeks post arrival for birds fed a control (Ctrl wk 19) or biochar supplemented diet (BC wk 19). a) Genus level redundancy analysis (RDA) plot demonstrating differential separation of microbial communities due to time (arrival vs 19weeks post arrival) with no differential separation between control (white) and biochar (black) fed birds; b) Richness (genus level); c) Evenness (genus level) d) Abundance of Proteobacteria and Firmicutes in birds at arrival, compared to dietary treatments at 19 weeks post arrival on farm. ***Significance at *P* < 0.001.

On Farm 1, birds arrived as 15-week-old pullets and were fed either control or biochar diets for a duration of 19 weeks. Samples were taken from birds on arrival (15 weeks of age) to the layer farm before being separated onto control and biochar diets. These pullets were exposed to the stresses of transport and adaptation to a new farm diet (control or biochar amended) and environment at the time of coming into lay. There were significant microbial community differences between birds at arrival and samples taken at 19 weeks post arrival, measured by Bray-Curtis based Adonis analysis at both phylum (*P* < 0.001) and genus level (*P* < 0.001). The microbial communities of birds fed either diet at 19 weeks post arrival however did not significantly differ (*P* > 0.05; Fig 3A). Microbial richness significantly increased at both genus (*P* = 0.0248; Fig 3B) and OTU level (*P <* 0.001) in samples from both control and biochar treatments at week 19 post arrival compared to that at arrival, however not at phylum level (*P* = 0.139), nor was there a difference between biochar or control fed birds at week 19 post arrival. Evenness was not significantly different between groups (arrival, control or biochar fed birds) at genus (*P* = 0.974, Fig 3C) or OTU (*P* = 0.898) levels, however it was significantly different at phylum level (*P* < 0.001).

**Fig 4:**
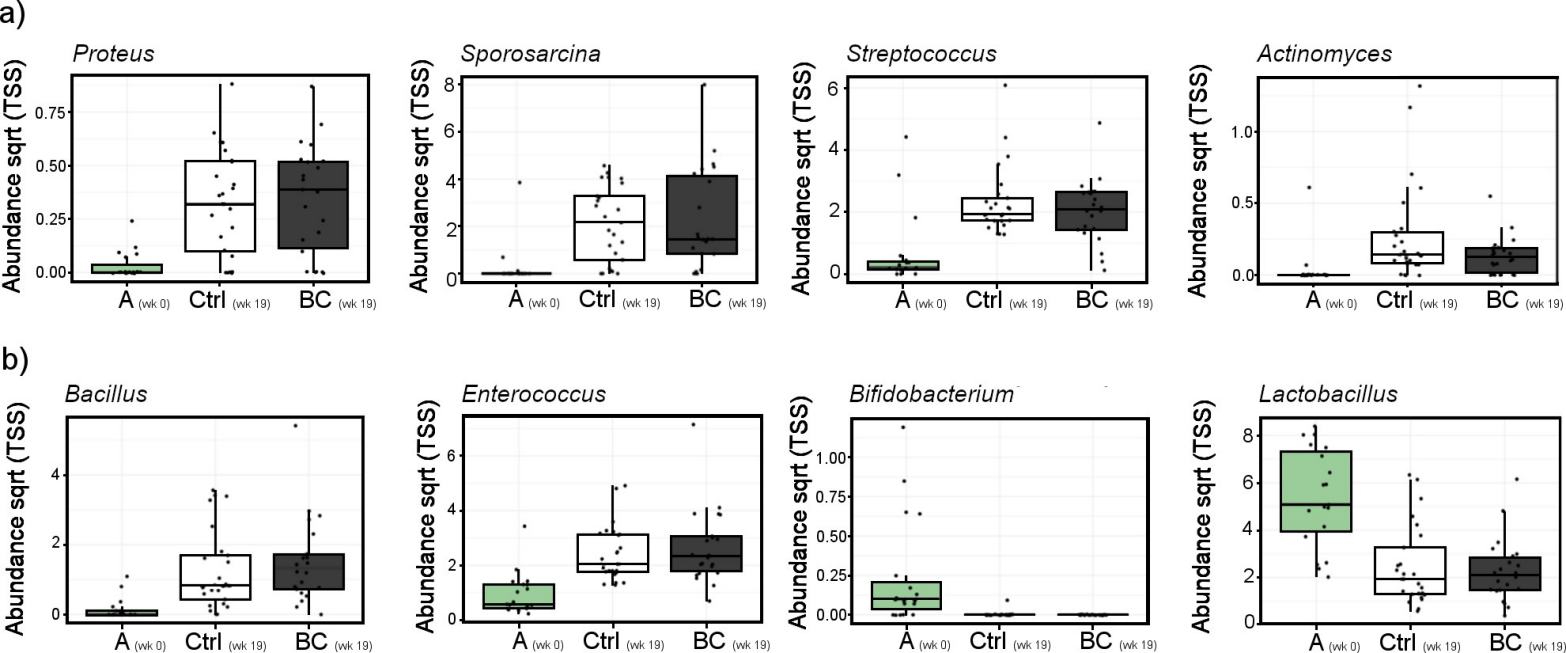
Microbial abundance in cloacal swabs of layer chickens on control and 2% w/w biochar amended diets for a period of 19 weeks (Trial 2) at arrival to farm (A wk 0), and 19 weeks post arrival for birds fed a control (Ctrl wk 19) or biochar supplemented diet (BC wk 19). a) Increases in pathogenic associated genera *Proteus*, *Sporosarcina*, *Streptococcus* and *Actinomyces*; b) Desirable genera including increases in *Bacillus* and *Enterococcus* and reductions in *Bifidobacterium* and *Lactobacillus*.

The major changes in the phyla present between control and biochar fed birds were decreased abundance of Firmicutes (R = -0.45; *P* < 0.001) and increased abundance of Proteobacteria (R = 0.34; *P* < 0.001; Fig 3D). Synergistetes were also significantly increased in abundance from arrival to 19 weeks post arrival (*P* < 0.001) but not between diets. The increase of Proteobacteria was accompanied by a rise in unknown *Enterobacteriaceae* over time in both biochar (*P* < 0.001) and control fed birds (*P* < 0.001). Blasting of this sequence identified it as more similar to uncultured bacteria of clay or soil origin than to *Enterobacteria*. After 19 weeks of treatment, *Gallibacterium* abundance was reduced below detection level in both the control birds as well as those fed biochar (R = -0.29; *P* = 0.003). Conversely, genera harbouring pathogenic species increased over time, including opportunistic pathogens *Proteus* and *Actinomyces*, known pathogenic *Streptococcus* and proposed pathogenic *Sporosarcina* (Fig 4A). Of the genera generally regarded as beneficial, both *Bacillus* and *Enterococcus* were increased, while decreases were seen in both *Lactobacillus* and *Bifidobacterium* over the 19 week period in both the biochar and control diets (Fig 4B).

### Trial 3—Farm 2

**Fig 5:**
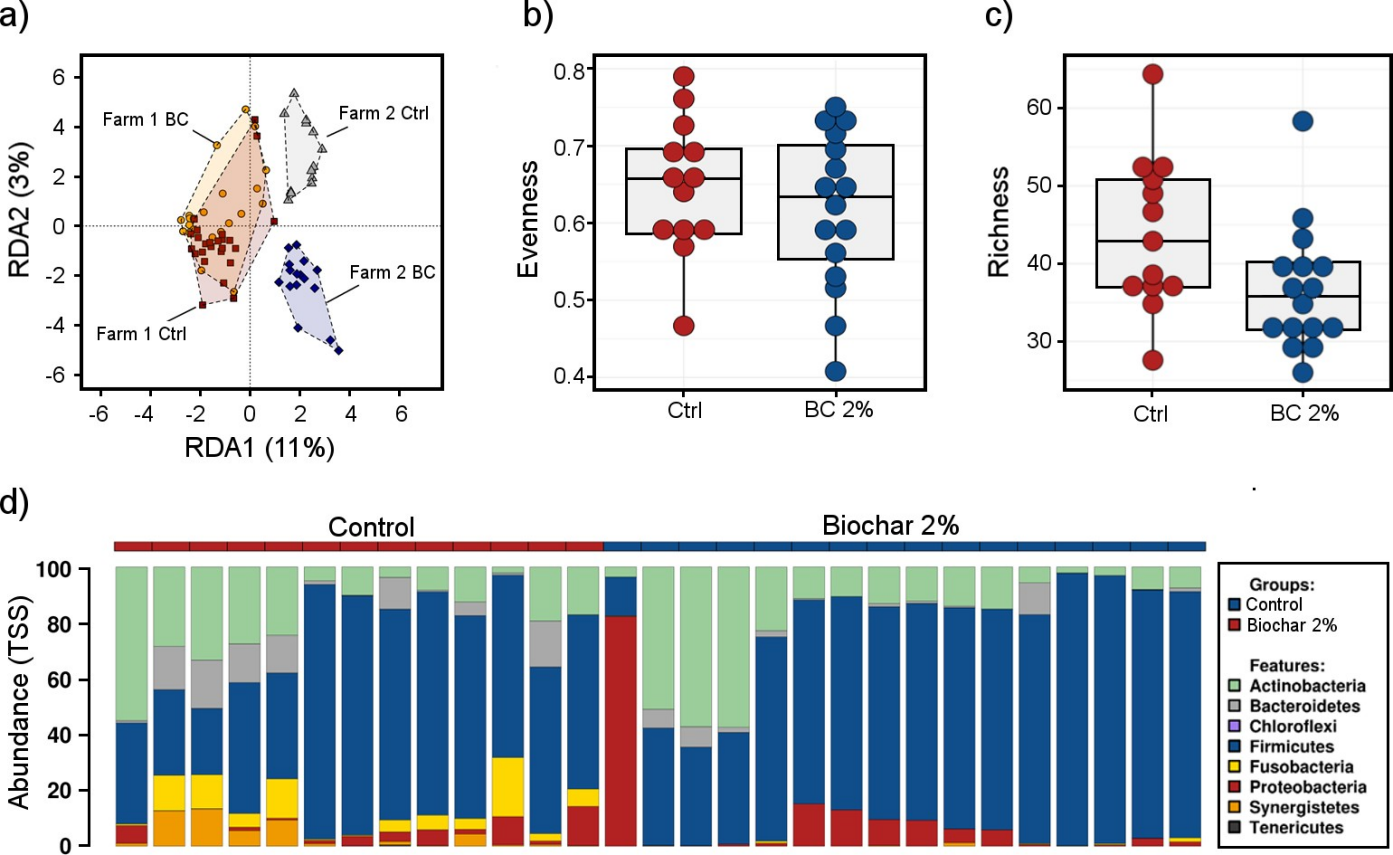
Microbial diversity in cloacal swabs of layer chickens fed either control (Ctrl) or biochar (BC) diets from hatch for a duration of 48 weeks. a) Redundancy analysis (RDA) plot demonstrating no differential separation of control (Ctrl) vs biochar (BC) fed birds on Farm1 and differential separation of control (Ctrl) vs biochar (BC) fed birds on Farm 2; b) Evenness (phylum) and c) Richness (phylum) indices for Ctrl vs BC fed birds on Farm 2; d) Relative abundance of the phyla present in control birds vs biochar fed birds on Farm 2.

Dietary treatment on Farm 2 (Trial 3) began on arrival of day-old chicks, in contrast to the previous two reported studies in which biochar supplementation commenced when the birds were 17 week old pullets in the Trial 1-dose dependent study, and as 15 week old pullets in the Trial 2-Farm 1 study. In Farm 2, where the birds were fed from hatch, community structure was significantly different as measured by Bray-Curtis Adonis for both phylum (*P* = 0.001) and genus (*P* = 0.005) levels. The RDA plot comparing Farm 2 against Farm 1 shows differential separation of birds fed either control or biochar diets from hatch, compared to Farm 1 where dietary treatments started at 15 weeks of age (Fig 5A), showing no differentiation. At the phylum level, both Evenness (*P* = 0.040; Fig 5B) and Richness (*P* < 0.001; Fig 5C) were decreased in the biochar supplemented birds, however only richness was reduced at the genus level (*P* = 0.037). Neither were different at OTU level.

**Fig 6:**
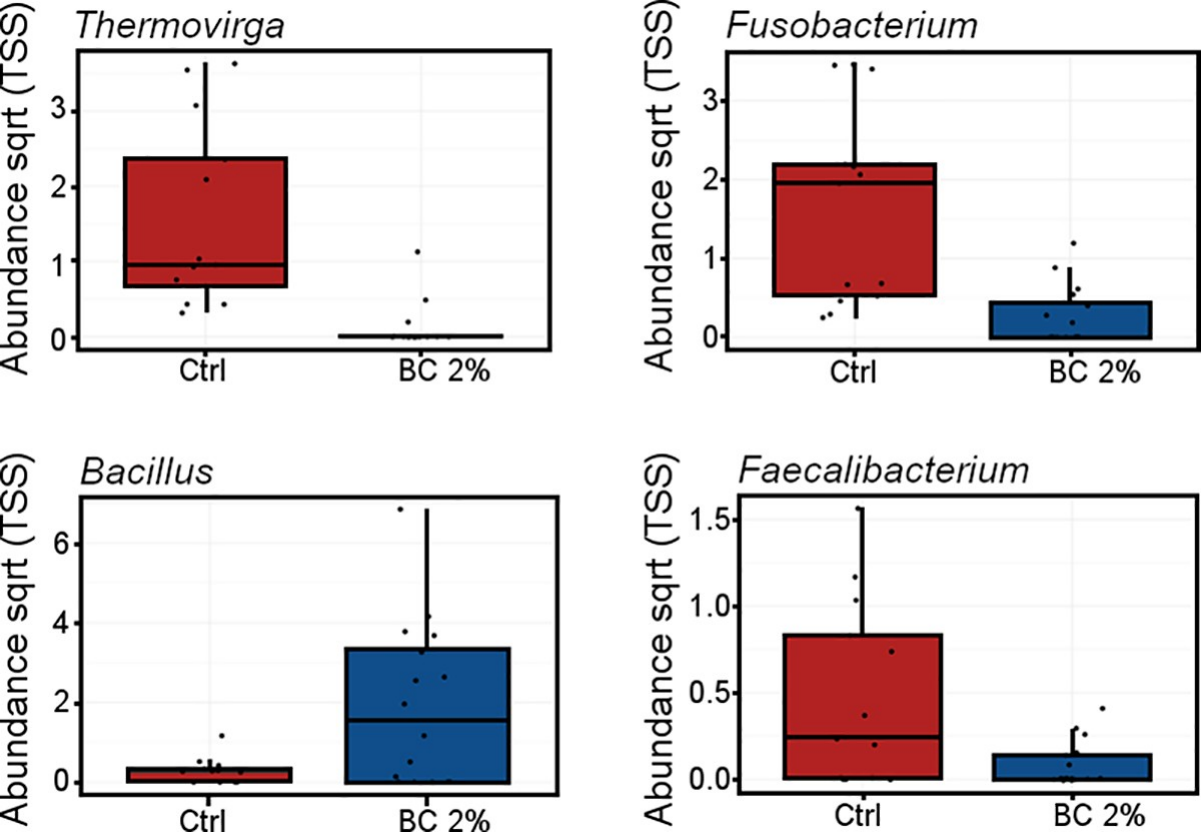
Differentially abundant genera from Farm 2 cloacal swabs including: *Thermovirga* (*P* = 0.001), *Fusobacterium* (*P* = 0.002), *Bacillus* (*P* = 0.006) and *Faecalibacterium* (*P* = 0.024).

The most abundant phyla across Trial 3 (Fig 5D) were Firmicutes (65.0%), Actinobacteria (18.7%), Proteobacteria (6.8%) Bacteroidetes (4.5%) Fusobacteria (3.2%) and Synergistetes (1.8%). The most abundant phyla were not significantly altered in abundance between biochar and control birds but rather rare phyla present; Fusobacteria (*P* < 0.001; 31.6-fold increase), Synergistetes (*P* = 0.001; 39.2-fold increase) and Bacteroidetes (*P* = 0.027; 3.4-fold increase) were in higher abundance in control birds compared to the biochar supplemented birds. Genera differentially abundant (Fig 6) within these phyla included *Thermovirga*, (*P* = 0.001) and *Fusobacterium*, both of which were reduced in biochar supplemented birds (*P* = 0.002). *Bacillus* was increased in biochar fed birds (*P* = 0.006) including the species *B*. *cereus* (*P* = 0.014) and *B*. *thermoamylovorans* (*P* = 0.018), conversely, beneficial *Faecalibacterium* was reduced in the biochar fed birds (*P* = 0.024).

### PCR investigation of pathogen reduction

PCR was used to detect the presence or absence of *Campylobacter hepaticus* (the causative agent of Spotty Liver Disease) in both biochar and control fed birds on Farm 1 and Farm 2. On Farm 1, carriage of *C*. *hepaticus* was detected in 50% of the 16 control birds tested (8/16 positive), and 7.7% in the biochar fed birds (1/13 positive), which was significantly different between the two treatments (*P* = 0.014). This result would have benefited from knowing the original *C*. *hepaticus* level when birds were transferred on farm as 15 week old pullets. Farm 2 reported increased mortality in the weeks preceding the 48 week sampling timepoint, which was suspected to be a Spotty Liver Disease outbreak. No antibiotics were administered during this time. There was a cumulative mortality of 16.5% in control fed flock and 10.9% cumulative mortality in the biochar fed flock by week 48 (week of sampling). PCR conducted on the DNA from the cloacal samples taken from Farm 2 at 48 weeks post hatch did not detect *C*. *hepaticus* in either control (*n* = 13) or biochar (*n* = 16) fed birds.

## Discussion

Results from the dose dependent study (Trial 1) indicate that the anti-pathogenic effects of biochar on intestinal microbiota via cloacal swabs were most beneficial in terms of impact on microbiota at inclusion rates up to 2% w/w, after which little additional benefit was seen, or reversed in some instances. Proteobacteria were significantly reduced in birds supplemented with biochar. Within this phylum, significant reductions were found for *Campylobacter* at 1% and 2% w/w inclusion, however this result was not correlated with supplementation level as the abundance was comparatively increased slightly at 4% w/w biochar inclusion. The reduction of *Campylobacter* load is a promising result from a public health perspective, as poultry are a known reservoir for *Campylobacter* and carriage is typically asymptomatic in chickens. *Campylobacter* infections are the global leading cause of acute gastroenteritis in humans, with 50–70% of infections attributed to consumption of contaminated chicken products [32]. Dose dependent reductions were also evident for the genus *Gallibacterium*, including *G*. *anastis*, a poultry pathogen that results in loss of production via decreased egg production and increased mortality [33]. *Gallibacterium* were detected in birds on Farm 1 at arrival, however it was not detected by week 19 post arrival in either the control or biochar fed birds and therefore not attributed to biochar inclusion in this case.

A commonality between the commercial farms was the detection of Synergistetes. In both instances, birds fed the biochar diets had a decreased abundance of this phylum, of which only the genus *Thermovirga* was detected. These are members of an unnamed division of taxa that Vartoukian et al. [34] termed ‘*Synergistes’* for the purpose of their review. They are anaerobic amino acid degraders, occurring naturally in niche environments and have been isolated from human and animal digestive tracts [34]. To our knowledge this genus has not previously been reported in chicken microbiota. Vartoukian et al. [34] conclude in their review that they are frequently detected in mucous membrane associated infections and expect that they will present in polymicrobial infections predominated by anaerobes and free amino acids. Increases in *Bacillus* were also detected with biochar treatments. While some strains are used as a probiotic, others can be problematic. Of the two strains provisionally identified in Farm 2, *B*. *cereus*, has been associated with food poisoning as well as production of tissue-destructive exoenzymes [35], and *B*. *thermamylovorans* has been identified as an emerging threat to the dairy industry as a contaminant due to its high thermal tolerance [36]. It must be noted however that these were detected at less than 2% abundance on average and in heathy, well performing birds.

Commercial farm environments are naturally more challenging to control than experimental settings. Farm 1 was characterised by an increase in Proteobacteria and a decrease in Firmicutes, which is generally considered a marker of dysbiosis and stress [37]. This occurred in both the control and biochar fed birds, with the increased richness indicating the birds had adopted a number of new species from the new farm environment. Increases in pathogenic genera in both the control and biochar fed birds included *Proteus*, *Actinomyces*, *Streptococcus* and the proposed pathogenic *Sporosaricna*. Conversely, beneficial genera such as *Lactobacillus* and *Bifidobacterium* were reduced in the microbiota of birds on the biochar amended diet. These time related changes in the microbiota may be due to accumulated stress on the birds (i.e. transportation, adaptation to a new environment, coming into lay and dietary changes) as both chronic and acute stressors have been shown to disrupt the composition of the microbiome in multiple species [38]. Alternatively, the increase in Proteobacteria may reflect the increase in unclassified *Enterobacteriaceae* in both treatment groups. Blast analysis identified this bacterium to be of a soil or clay origin opposed to a ‘bad’ Proteobacteria, which may have been responsible for the decline of the initially abundant *Lactobacillus*. Either scenario may have mitigated the potential benefits of biochar on pathogen control on Farm 1 and highlights the challenge in translating experimental results into commercial environments.

An issue for further consideration in determining the efficacy of an in-feed additive, particularly on farm, is the timing of dietary supplementation. The dose dependent trial and Farm 1 began biochar supplementation with birds at 17 and 15 weeks old respectively, a common age to acquire a new layer flock commercially. In these cases, the community structures were not significantly different between the biochar and control fed birds in each trial, despite the biochar being from two different sources. At Farm 2, in which microbial communities were distinct in biochar and control fed birds, treatments were imposed at receival to the farm as day-old chicks. In chickens, major microbiota colonisation occurs immediately during and after hatch, which is influenced by the microbiota on the eggshell, in the environment, and in the diet [39]. This occurs with concurrent development of systemic immune competence in chickens particularly during the first 2 weeks post hatch [40]. Therefore, the community separation of microflora established at hatch in Farm 2 is not surprising, however does this equip birds with differential immunological competency? This would be particularly relevant to investigate in birds that will undergo a level of environmental stress nearing point of lay as occurred in Farm 1, as Farm 2 reared their own pullets onsite therefore the birds did not undergo transportation or environmental change stress experienced by the Farm 1 flock.

Additional conditions differing between the two farm trials included the age of the flocks at the time of sampling, and the source of biochar which differed between the farm trials (Trial 2 and 3) and the dose dependent study (Trial 1). Future studies should seek to optimise the biochar dose for a standard biochar source, as properties will differ between sources. Critical assessment of the impact of age of birds for starting biochar additives, and effects of biochar on various health challenges would be beneficial. For layer chickens, further evaluation of feed efficiencies and egg quality aspects, including sensory, will also be crucial for further extension of biochar feeding to poultry. Spotty Liver Disease is a re-emerging disease in the Australian egg industry resulting in significant productivity reductions and high incidences of mortality [41]. The causative pathogen was identified and named by members of our team in 2016 as *Campylobacter hepaticus* [41]. *C*. *hepaticus* is believed to infect via the fecal-oral route and can colonise the gut. Birds are most vulnerable to development of Spotty Liver Disease whey they are entering peak lay and it is hypothesised that physiological stress or other changes in the homeostatic state of the gut are predisposing factors for the translocation of *C*. *hepaticus* from the gut to the liver and gall bladder, resulting in clinical disease [31]. Samples from the two farms were tested using PCR for the presence of *C*. *hepaticus* which was confirmed Farm 1. During the experimental period, Farm 2 suffered a widespread clinical outbreak of Spotty Liver Disease, with the producer reporting higher mortality in the control birds. Random sampling on farm post the clinical outbreak were all negative for *C*. *hepaticus*. Samples collected from Farm 1 were also screened for *C*. *hepaticus*, biochar fed birds had carriage of *C*. *hepaticus* at 7.7%, compared to 50% of the control birds tested and confirmed as positive. The mechanism by which biochar may have reduced *C*. *hepaticus* carriage (i.e. timing of feed, differential immune competence, biochar properties, less carriage to begin with etc.) and reportedly reduced mortality during the clinical outbreak is unclear and requires further investigation with much higher numbers sampled and analysed for carriage. These preliminary results however indicate that biochar is a promising candidate for further investigation and optimisation as an alternative to antibiotics for pathogen control.

In summary, dietary supplementation of biochar could be a promising alternative to sub-therapeutic antibiotics, particularly for layer and organic enterprises. The effect of biochar against pathogenic bacteria was found to be most beneficial at 2% w/w although the possible mechanisms for selective effects on microbiota require further investigation. Poultry pathogens such as *Campylobacter*, *Gallibacterium anatis* and *C*. *hepaticus* were found to be significantly reduced in biochar fed birds. The results of the current studies also indicate differential effects of feeding from hatch vs supplementation at later time points, suggesting that early post-hatch feeding alters the community structure to a greater extent that at lay imposed treatment. The at-hatch treatment also involves an impact on microbial colonisation concurrent with early immune development in chicks. Additional investigation into the mechanism of biochar function, particularly against *C*. *hepaticus*, as well as assessment of any long-term risk to flock health, including potential long-term toxicity is recommended.
